# From Regeneration to Analgesia: The Role of BPC-157 in Tissue Repair and Pain Management

**DOI:** 10.3390/ijms27062876

**Published:** 2026-03-22

**Authors:** Claire Yuan, Ariana Demers, Victor Silva-Ortiz, Jamal J. Hasoon, Woojin Lee, Karan Dave, Kasra Amirdelfan, Harold W. Burke, Paul J. Christo, Christopher L. Robinson

**Affiliations:** 1 Department of History and Philosophy of Science, Christ’s College, University of Cambridge, Cambridge CB2 1TN, UK; 2 Joint Vitality Institute, Sonora, CA 95370, USA; 3 Pain Management Department, Hospital Zambrano Helión, Monterrey, Nuevo León 66278, Mexico; 4 Department of Anesthesia, Critical Care, and Pain Medicine, McGovern Medical School, UTHealth, Houston, TX 77030, USA; 5 Department of Anesthesiology, Warren Alpert Medical School, Brown University, Providence, RI 02903, USA; 6 Aurora Life, Grand Cayman KY1-1209, Cayman Islands; 7 Boomerang Healthcare, Walnut Creek, CA 94598, USA; 8 Division of Pain, Department of Anesthesiology and Critical Care, The Johns Hopkins University School of Medicine, Baltimore, MD 21287, USA

**Keywords:** BPC 157, regenerative medicine, pain management

## Abstract

Body Protective Compound-157 (BPC-157) is a synthetic pentadecapeptide derived from gastric proteins that has demonstrated notable reparative and anti-inflammatory properties across diverse preclinical models. Experimental evidence reveals that BPC-157 supports angiogenesis, collagen synthesis, fibroblast activity, and modulation of nitric oxide pathways, contributing to enhanced healing of muscle, tendon, ligament, bone, and gastrointestinal tissue. Studies also report reduced inflammatory cytokine activity, improved microvascular integrity, and beneficial effects on pain modulation through peripheral and dopaminergic mechanisms. Although animal data indicate favorable safety and pharmacokinetics, human research remains limited to small pilot studies investigating musculoskeletal pain, interstitial cystitis, and intravenous administration, all suggesting potential therapeutic value without reported major adverse effects. However, inconsistent preparation standards, limited clinical validation, and regulatory restrictions underscore the need for rigorous controlled trials. BPC-157 remains a promising candidate for regenerative medicine, yet comprehensive evaluation is required before clinical translation can be recommended.

## 1. Introduction

The push to find effective and biologically suitable interventions in pain management has become a priority in recent years. This is particularly pressing given concerns around the adverse effects of pharmacotherapies and the emerging obsolescence of opioid analgesics. Regenerative medicine and related biomaterials present a potential alternative, having recently shown positive results in improving effectiveness and biocompatibility by directly acting on cellular and molecular pathways necessary for wound healing and tissue regeneration [1,2].

Researchers have dedicated significant efforts towards developing safe and effective therapies to promote healing while introducing limited to no adverse effects. The focus has been particularly directed towards peptides due to their natural occurrence. Biologics derived from these peptides are expected to not only reduce risks and adverse effects but also improve treatment efficacy through efficient metabolism and precise molecular targeting [3,4,5]. Several regenerative modalities have already demonstrated varying levels of therapeutic success in treating back pain due to lumbar degenerative disc disease, knee osteoarthritis, tendinitis, rotator cuff injury, and chronic neuropathic pain due to nerve inflammation [1,6,7,8]. Further, some biologics—including platelet-rich plasma (PRP) and bone marrow aspirate concentrate (BMAC)—have shown potential for dual-action therapies, featuring not only direct action on wound healing pathways but also antibacterial and antifungal properties to support an effective and efficient recovery [5,9,10,11].

This review will focus on Body Protective Compound-157 (BPC-157), a synthetic peptide isolated from gastric juice [12]. First introduced in 1993, BPC-157 is a fragment consisting of 15 amino acids derived from proteins that promote angiogenesis, neuromuscular stabilization, fibroblast activity, and endothelial and muscular repair [12,13,14,15]. It may also contain anti-inflammatory properties, promoting healing mechanisms [12]. BPC-157 has significant implications for both sports and pain medicine for the treatment of musculoskeletal injuries, particularly those that result from muscle fiber tears [13]. Additionally, the therapy has shown significant promise in healing injuries related to ligaments, tendons, and bones [16]. In animal models, treatment of wound sites with BPC-157 demonstrated improved results compared to the presence of only common factors necessary for wound healing, including EGF, FGF, and VEGF, with various biologically compatible carriers [17]. Still, numerous translational gaps remain, and the peptide will require further investigation to ensure proper and safe use in everyday clinical settings. Despite this, the public appeal of BPC-157, particularly within athletic circles, has placed pressure on clinicians to reckon with the rising popularity of poorly regulated, illicit use of this peptide therapy.

### Preparation

While BPC-157 can be naturally extracted from gastric secretions, it is often chemically synthesized in lab settings for research applications. BPC-157 may be prepared using solid-phase peptide synthesis (SPPS). In SPPS, amino acids are added stepwise onto a resin carrier, with protecting groups such as Boc or Fmoc and the use of coupling reagents like DIC/HOBt and HATU to activate the carboxyl group [18]. The fully assembled peptide is subsequently cleaved from the resin for further purification and processing [19]. Purification techniques include high-performance liquid chromatography (HPLC) to eliminate incomplete chains or other unintended products, allowing for approximately 99% purity of BPC-157 [20].

Once fully prepared and purified, the peptide may be lyophilized into a powder form for storage and shipment, thereby preventing degradation prior to use [20]. Though such methods are intended to allow for easier maintenance and access for research purposes, they also facilitate illicit access to BPC-157, circumventing the need for extensive equipment and invasive procedures for administration.

## 2. Mechanisms of Action in Pain Modulation and Healing

Wound healing occurs in multiple overlapping phases: coagulation and hemostasis, inflammation, proliferation, and wound remodeling [21]. During this process, a wide variety of proteins and signaling molecules enable and promote tissue repair and regeneration [22]. In the immediate aftermath of injury, blood vessels constrict, initiating the coagulation cascade to reduce blood loss. For hemostasis, platelets degranulate and release α-granules to secrete growth factors (GF) [22]. Macrophages, fibroblasts, and endothelial cells are further attracted to the wound site to begin the healing cascade, forming a clot composed of fibronectin, vitronectin, fibrin, thrombospondin, and von Willebrand factor [22].

The second stage of wound healing is further divided into two phases: (1) early inflammation, which lasts 1–2 days and begins with the complement system, and (2) late inflammation, which lasts for the following 1–2 days and involves the attraction of blood monocytes to the wound to become tissue macrophages [22]. These regulate cell repair by producing GFs for smooth muscle and endothelial cell proliferation and extracellular matrix (ECM) production, as well as by working as phagocytic cells [22].

The proliferation phase begins approximately three days following initial injury and lasts 2–4 weeks [22]. In this third phase, fibroblasts migrate to the wound site to generate hyaluronan, collagen, fibronectin, proteoglycans, and various matrix proteins to generate the new ECM [23]. Further, granulation tissue forms, when the basement membrane of the parent vessel is degraded, forming a capillary spout. Endothelial cells then migrate, proliferate, and mature into capillary tubes. Finally, epithelialization begins with the modulation by GFs [22]. Wound healing concludes with remodeling, including scar tissue maturation, which occurs over the course of multiple weeks. During this time, collagen is regularly formed and degraded by metalloproteinases at the site of the wound [22,24].

There are a multitude of steps and factors at play in wound healing, of which BPC-157 is only one. Studies have suggested that BPC-157 supports the wound healing process by boosting angiogenesis, stimulating collagen production, promoting cell migration and proliferation, and preventing excessive and chronic inflammation (uncontrolled inflammation post-injury may result in further tissue damage) [12,25,26]. This is due, in part, to BPC-157 action in modulating the nitric oxide (NO) system [27]. Further, BPC-157 activates pathways like the extracellular signal-regulated kinases (ERK1 and ERK2) and vascular endothelial growth factor (VEGF), which are also crucial aspects of wound repair and regeneration, resulting in increasing use of the biologic in preclinical models for burns, other skin injuries, and various soft tissue damage (Table 1) [12,21]. Still, the data available for specific BPC-157 action in wound healing is limited. As such, note that some of these effects are still extrapolative rather than directly proven.

**Table 1:** Key cellular and molecular components of wound healing and BPC-157 action.

Type	Role in Wound Healing	Reported Effect of BPC-157
Cells		
Platelets	Formation of thrombin [28]	May protect platelet function in animal models [29]
Leukocytes	Phagocytose bacteria, foreign particles [30]	Possible immunomodulatory effect; normalize inflammation [31]
Growth factors		
VEGF	Stimulate formation of blood vessels Promote granulation tissue angiogenesis [32]	Upregulates VEGFR2 expression in endothelial cells for animal models [33]
Plasma proteins		
Albumin	Maintain fluid balance Transport growth factors [34]	Indirect effects via broad cyto- and hepato-protection [35] (indirect evidence, hypothesized)
Other		
Inflammatory cytokine modulation	Regulates inflammatory phase [26]	Reduced inflammatory markers [36]
Nitric oxide (NO) system	Vasodilation Endothelial homeostasis Inflammation modulation [27]	Modulates NO signaling Improves endothelial function [27]
Collagen synthesis, ECM remodeling	Matrix deposition Scar maturation	Increased collagen deposition Improved organization in burn, tendon healing [16,17,37]

## 3. Preclinical Models

Multiple animal model studies have investigated the healing properties of BPC-157 in vivo and in vitro (Table 2). This paper attempts to provide an overview of the existing preclinical literature landscape without editorial selection.

Preclinical studies suggest that BPC-157 promotes growth hormone receptor expression, may enhance pathways involved in angiogenesis and cell growth, and reduces inflammatory cytokines [25]. BPC-157 has also shown strong antioxidant properties, and such enzymes play regulatory roles in muscle regeneration [3].

Studies have indicated that BPC-157 supported wound healing in the esophagus, gastrointestinal tract, stomach, and duodenum in acute and chronic injury cases compared to controls in animal models [17,38]. In vivo and in vitro, BPC-157 promotes blood vessel density and improves ischemic muscle blood flow, therefore supporting the biologic’s positive impact on angiogenesis [33]. Of note, there was particular histological evidence of with increased expression of vascular endothelial growth factor receptor 2 (VEGFR2) and upregulation of growth hormone receptors [13]. Further, BPC-157 improved medial collateral ligament (MCL) healing in rats, including the expression of early growth response 1 (EGR1), a transcription factor implicated in cell growth, differentiation, and inflammation [37]. Given either intraperitoneally, as a local topical application, or orally, the therapy was shown to promote muscle and transected tendon healing [15,37].

Multiple studies have also reviewed the role of BPC-157 in skin wound and burn healing. In a rat model of an alkali skin burn injury, topical application accelerated wound closure and promoted granulation tissue formation, re-epithelialization, and dermal remodeling 2–3 weeks following injury (compared to typical burn healing, which can require several months) [39]. Similar results were noted by several other studies, with topical BPC-157 creams also decreasing inflammation and edema, improving collagen deposition, and angiogenesis [40]. Though most commonly and widely tested for skin or soft tissue injuries, BPC-157 has also shown potential in promoting healing in bone injury. In rabbits, BPC-157 was found to have osteogenic effects in healing segmental bone defects. Animals that received the therapy showed significantly improved healing after local delivery of bone marrow or autologous cortical graft compared to saline-treated controls [41]. Moreover, BPC-157 may also be considered for use across a variety of conditions, including congestive heart failure, colitis ischemia, catalepsy, ulcers, liver protection, and Parkinson’s disease [19,42,43,44,45].

In addition to direct or indirect intervention in various tissue, bone, and other disorders, BPC-157 may influence pain as well. In rats, available data suggest the local application of BPC-157 at surgical incision sites significantly raised the pain threshold at early time points hours following injury, but the reduced sensitivity effect was lessened by 7 days post-surgery [46]. Researchers posited that the antinociceptive effect of the biologic may occur through an anti-inflammatory mechanism that results in short-lived alleviation rather than a centrally mediated analgesic effect [46]. Further studies have indicated similar results in formalin pain models in rats, with BPC-157 decreasing flinching responses during acute phase 1 but not persistent phase 2 pain [47].

**Table 2:** Selected preclinical models of the wound healing and pain management actions of BPC-157.

Study	Model and Design	Key Findings
Huang et al. [39]	Rat, alkali skin burns (*n* = 10 per group) Topical application	Promoted wound closure, tissue formation 18 days post-wound
Mikus et al. [40]	Mouse, thermal burns (*n* = 10 per group) Topical application	Improved collagen deposition and reduced edema
Cerovecki et al. [37]	Rat, MCL damage (*n* = 10 per group) Intraperitoneal, oral, topical	Improved muscle repair, EGR1 expression
Hsieh et al. [33]	Rat (in vivo, in vitro), ischemia (*n* = 6 per group) Intraperitoneal injection	Upregulated VEGFR2, promoted angiogenesis
Keremi et al. [36]	Rat, periodontitis (*n* = 7 per group) Intravenous injection	Anti-inflammatory action
Šebečić et al. [41]	Rabbit, segmental bone defect (*n* = 12 per group) Percutaneous, intramuscular	Improved local osteogenic effects
Park et al. [47]	Rat, formalin-induced (*n* = 7 per group) Intraperitoneal injection	Reduced acute pain phase behavior, no chronic phase effect
Jung et al. [46]	Rat, incisional pain (*n* = 4–8 per group) Intraperitoneal injection	Increased early pain threshold, effect diminished by 7 days

Animal studies have suggested that BPC-157 exhibits anti-inflammatory effects, with chronic dosing particularly potent against ligature-induced periodontitis in rats [36]. Notably, BPC-157 has been seen to counteract inflammatory and non-inflammatory pain in mice, and the biologic may interact with the opioid system through dopaminergic pathways to influence sensitivity to pain [48]. Therefore, BPC-157 has the potential to serve as an alternative for traditional pharmacotherapies, including steroid medications.

Though further studies are needed for full characterization, some initial reviews of the pharmacokinetic properties of BPC-157 reported linear characteristics with 14–19% bioavailability in rats and 45–51% in beagle dogs [20]. Based on a prototype BPC-157, the half-life of the biologic in animals appears to be less than 30 min [20]. Still, such data—on bioavailability and other results—across species are limited [3]. Few robust pharmacodynamics and pharmacokinetic studies exist, highlighting the need for further investigation to confirm the efficacy, indications, and safety profiles.

In the available preclinical data, no adverse effects were observed across multiple organ systems [25]. In animal models, BPC-157 was metabolized into small peptide fragments to enter normal amino acid metabolism and subsequent excretory pathways [20]. Preclinical toxicity studies in mice, rabbits, dogs, and rats indicated that both single- and multi-dose studies did not induce significant adverse effects, though some mild local irritation manifested [49]. Further, in rats, BPC-157 did not show any teratogenic effects based on comparisons of the number and condition of fetuses, uterine and placental weight of the pregnant rats, and assessment of various other fetal and maternal malformations [49]. Such findings support the biochemical supposition that BPC-157 may present a promising therapeutic avenue to avoid the potential pitfalls of existing biologics.

Across preclinical data, multiple methods of BPC-157 administration are used, and the reported efficacy appears to vary modestly. Topical applications in cutaneous injury and burn models consistently demonstrated accelerated wound closure and improved healing, suggesting that local administrations may be sufficient for superficial injury sites [39,40]. Intraperitoneal injections also showed positive effects for ischemia, incision site pain, and formalin-induced pain, heightening pain thresholds and promoting wound repair [33,46,47]. Oral administration and percutaneous injection were also studied in some applications [37,41]. These findings collectively indicate that BPC-157 has the potential to promote wound healing and analgesic effects through multiple modes of administration, though certain methods may be better suited for specific injury types depending on the need for local optimization or systemic use, for instance.

However, it is also necessary to note that the heterogeneity of models and experimental designs pose challenges to the generalizability of such findings. Further studies are needed to validate the results reviewed in this section.

## 4. Limited Human Data

Despite increasing off-label use in pain management, particularly in sports medicine, BPC-157 has not been well studied for use in humans, with only a small handful of studies featuring small sample sizes (Table 3) [12]. This section aims to provide an overview of the existing data, including a study that was ultimately not published. A retrospective study of 12 patients found that intraarticular injection of BPC-157 for chronic knee pain produced relief for more than 6 months for 11 participants, supporting preclinical findings that BPC-157 can promote musculoskeletal healing [25,50]. However, in addition to a small sample size, the retrospective chart review nature of this study resulted in varied patient follow-up. Four patients also received a combination of BPC-157 with TB4, a naturally occurring protein fragment that is important for tissue repair, regeneration, and wound healing. Via survey, three reported significant improvement, while one had no pain relief [50]. Researchers concluded that intra-articular injection of BPC-157 may help relieve multiple types of knee pain, though they recommend further investigation with the aid of magnetic resonance imaging (MRI) scans [50].

**Table 3:** The limited studies of BPC-157 action in humans.

Study	Design	Findings
Lee and Padgett [50]	Retrospective chart review (*n* = 12) of chronic knee pain patients	Knee pain relief with variable dosage intra-articular injection (combination therapy involved)
Lee et al. [51]	Pilot study (*n* = 12) of interstitial cystitis patients	Interstitial cystitis symptom and pain relief with 10 mg intravesicular injection No adverse effects
Lee and Burgess [52]	IRB-approved study (*n* = 2), safety profile in healthy adults	No adverse effects with ≤20 mg IV infusion, rapid clearance rate

A pilot study on the effects of BPC-157 in patients with interstitial cystitis revealed potential for the use in bladder pain, with 10 of 12 patients reporting total symptom relief after one 10 mg intravesical injection [51]. The remaining 2 patients rated their symptom resolution at 80%. Data was collected by administering the Global Response Assessment questionnaire [51]. Further, researchers reported seeing no adverse effects [51]. Still, as with other human clinical data, this study remains limited by a small sample size; so, it is necessary to regard results with some caution.

While very limited human data exist for BPC-157 in humans, some attention has been dedicated towards its safety and pharmacokinetic profile. A small (*n* = 2) study was performed to assess intravenous BPC-157 safety in humans at up to 20 mg [52]. Researchers found no measurable impacts on the patients’ heart, kidneys, thyroid, liver, or blood glucose via obtaining blood work and vital signs, and neither patient reported adverse side effects when asked at each appointment [52]. Plasma BPC-157 concentrations returned to baseline levels within 24 h, supporting existing pharmacokinetic understanding of the biologic’s quick bodily clearance [52]. This pilot study is the only characterization of half-life in humans, therefore indicating a shortage of robust peer-reviewed data in this area [12].

As with preclinical studies, the limited BPC-157 studies in humans also investigated various methods of biologic administration, each of which indicated some potential positive effects. These findings similarly suggest that each method of application may be preferable for particular injury types.

Despite the encouraging data, further studies are necessary to fully assess the safety and efficacy of BPC-157 therapy in humans. Notably, much of the data suffer from small sample sizes, lack of appropriate and broad-ranging controls, and insufficient randomization. Additionally, in 2015, a phase I clinical trial was conducted on 42 patients to study pharmacokinetics and safety. Unfortunately, the results submission and publication were not published for unknown reasons [3,53]. Therefore, additional research should strive towards incorporating a larger patient population, as well as the inclusion of validated pain scales and long-term safety monitoring. An expanded base of research that addresses the aforementioned limitations will provide a stronger understanding of the biologic’s potential benefits and risks within humans.

## 5. Safety, Regulatory, and Ethical Considerations

It is notable that a considerable portion of the current evidence related to BPC-157 arises from a single research group. This highlights the continued and pressing need for further research in order to generate a diverse and deep base of understanding for application. Further, independent validation of existing studies is necessary.

Moreover, the lack of human randomized controlled trials investigating BPC-157 has resulted in researchers urging caution for its use in clinical settings, particularly as a sports enhancement or lifestyle maintenance measure [13,54]. Stable in water and gastric fluid, the peptide has the potential to reduce the required administration frequency due to enhanced biostability and bioavailability [55]. Nonetheless, concerns remained with regard to stability in vitro, limited application sites, and the necessity of biocompatible carriers [56].

Very limited safety information is available for BPC-157 therapy in humans, and despite the positive results of animal models and a few human studies, anecdotal evidence from the use of BPC-157 has raised concerns. Anonymous online user narratives reported injection site swelling and pain, anxiety, heart palpitations, weakness, depression, loss of appetite, fatigue, insomnia, joint pain, drowsiness, and anhedonia [25]. It is important to recognize that these data are unconfirmed and ought to be treated cautiously, placed low in the evidence hierarchy. Nonetheless, it is worth considering how these complaints may manifest due to the unregulated production and variable dosing of BPC-157, amongst other causes [25]. Furthermore, BPC-157 may cause pain and/or necrosis upon injection into the subject alongside normal saline or other aqueous solutions [3]. As such, there remains a need for further characterization of the proper preparation and administration of BPC-157 as a pain management and/or sports medicine therapeutic.

While all peptide therapies can be applied via injection to the injury site, BPC-157 has also shown preclinical effectiveness through topical or oral applications. Further, unlike therapies such as PRP and BMAC that must be autologous in nature to be effective, thereby making it cost- and labor-intensive, BPC-157 that can be manufactured, distributed, and applied ubiquitously [2]. As such, most peptides and other orthobiologics can only be accessed in clinical settings under supervision, unlike BPC-157, which can be obtained through gray channels such as unregulated or semi-regulated distribution pathways [25].

Though BPC-157 has been swept into the growing enthusiasm around peptide therapies—in and outside of formal clinical settings—significant safety, ethical, and legal concerns around its uses remain. Despite biologic progress and potential for wound healing, this has largely not translated to official policy and regulation settings. As of the most recent 2025 World Anti-Doping Agency (WADA) list of prohibited substances, BPC-157 remains a prohibited substance [57]. Despite being banned from use in professional sports, BPC-157 is gaining attention from athletes and those in sports medicine [25]. Moreover, it does not have U.S. Food and Drug Administration (FDA) approval [25]. BPC-157 has also become quite popular in mainstream Hollywood and other internet influencer circles, with online “wellness clinics” and celebrities promoting such unapproved peptide drugs as wellness, fitness, and youth hacks [58,59,60,61]. This has only raised further clinician and policymaker concerns surrounding the safety of biologics like BPC-157 circulating on the market [58,59].

## 6. Conclusions

BPC-157 has significant potential as a biological therapy for a variety of tissue injuries, bone defects, and other conditions. In particular, BPC-157 promotes wound healing through interactions with cells, peptides, and signaling molecules during the tissue regeneration process. Studies have also indicated that it has action to mitigate inflammation and inflammatory pain. Moreover, due to its reparative effects, BPC-157 offers a possible alternative for pain relief [50].

However, significant concerns remain, most notably due to the lack of human data related to BPC-157 efficacy and safety. While animal models have suggested that BPC-157 is effective in a variety of pain management and wound healing applications without adverse effects, anecdotal evidence of unregulated uses of the biologic raises concerns about how well these findings may extend to human applications. As such, further research is necessary to fully characterize the efficacy, safety, and pharmacokinetics of BPC-157 as a viable therapeutic intervention. Randomized controlled trials are needed for establishing human efficacy and toxicology.

Further, priority should be placed on well-defined, high-prevalence indications based on existing preclinical data—in particular, to independently verify findings, provide standardization in dosing and formulation, and further assess safety. Trial designs ought to be strongly considered as well, with a focus on including validated pain scales, rather than solely relying on patient narratives, as well as measuring functional outcomes.

Moreover, standardization and regulation of BPC-157 preparation is necessary to prevent contamination and adverse effects, particularly in settings where access to controlled clinical sites is limited. Currently, there are no standardized guidelines around dosing and protocols, particularly for potential use in humans, marking an important hurdle to be cleared before BPC-157 can move toward official sanctioned use in the clinical and public spheres [20]. As mainstream attention continues to linger on peptide therapies for athletic recovery, youth maintenance, lifestyle care, and more, the need for exhaustive study and careful regulation grows increasingly pressing. Despite the lack of standardization and further studies, BPC-157 and other peptides, once researched more extensively, may offer patients suffering from injuries another avenue that aids in joint preservation, tissue regeneration, and improved pain relief.

## Data Availability

No new data were created or analyzed in this study.

## Abbreviations

BMAC	Bone marrow aspirate concentrate
BPC-157	Body Protective Compound-157
Boc	tert-butoxycarbonyl
DIC	Diisopropylcarbodiimide
ECM	Extracellular matrix
EGF	Epidermal growth factor
EGR1	Early growth response 1
ERK1/2	Extracellular signal-regulated kinase 1/2
FGF	Fibroblast growth factor
Fmoc	9-fluorenylmethyloxycarbonyl
GF	Growth factors
HATU	Hexafluorophosphate Azabenzotriazole Tetramethyl Uronium
HOBt	1-Hydroxybenzotriazole
HPLC	high-performance liquid chromatography
MCL	medial collateral ligament
MRI	Magnetic resonance imaging
NO	Nitric oxide
PRP	Platelet-rich plasma
SPPS	solid-phase peptide synthesis
TB4	Thymosin Beta-4
VEGF	Vascular endothelial growth factor
VEGFR2	Vascular endothelial growth factor receptor 2
WADA	World Anti-Doping Agency
